# COVID-19 Vaccination: Does It Alter Postoperative Mortality and Morbidity in Hip Fractures?

**DOI:** 10.7759/cureus.32688

**Published:** 2022-12-19

**Authors:** Nuthan Jagadeesh, Jabez Gnany, Sachindra Kapadi, Nidhin Koshy, Debbie Shaw

**Affiliations:** 1 Trauma and Orthopaedics, Vydehi Institute of Medical Sciences and Research Centre, Bengaluru, IND; 2 Trauma and Orthopaedics, Wrightington Wigan and Leigh NHS Foundation Trust, Wigan, GBR; 3 Orthopaedics and Traumatology, Royal Infirmary of Edinburgh, Wigan, GBR

**Keywords:** morbidity, mortality, covid vaccination, covid-19, hip fractures

## Abstract

Introduction

The primary aim of our study was to evaluate the impact of coronavirus disease 2019 (COVID-19) vaccination on mortality in patients with hip fractures by comparing it to those who are unvaccinated. Our secondary objective was to compare the impact on postoperative morbidity parameters like length of hospital stay and complications.

Methods

A total of 619 hip fractures were divided into the 'Vaccinated group' consisting of 300 patients who had COVID-19 vaccination and the 'Unvaccinated group' consisting of 319 patients who were not vaccinated. Patient demographic variables, Nottingham hip fracture score, American Society of Anesthesiologists (*ASA*) grade, type of anaesthesia used, and Charlson Comorbidity Index were collected. Our primary outcome measures were 30- and 90-day mortality. Secondary outcome measures included postoperative complications like thromboembolic complications, cardiac, and respiratory complications, etc. Mortality among the COVID-positive patients was also compared between the groups.

Results

Thirty-day postoperative mortality was higher in the unvaccinated group at 13.2% than in the vaccinated group at 5.3%. A similar increase in 90-day mortality was also observed in the unvaccinated group at 24.8% when compared to 14.7% in the vaccinated group(p<0.001). Despite having a higher baseline prevalence of cardiac comorbidities, the Vaccinated group had fewer post-operative cardiac, thromboembolic, and neurological complications (p>0.05). Moreover, electrolyte imbalance and AKI were also seen in fewer patients in the vaccinated group compared to the control group (p<0.05). Furthermore, it was found that among the vaccinated patients who did have perioperative COVID-19 infection, the 30-day mortality was significantly lower (10%) compared to the control group (31.2%) (p<0.001). Similarly, 90-day mortality was significantly lower (25%) compared to the control group (43.75%) (p<0.001).

Conclusion

Vaccination against COVID-19 independently reduced mortality and morbidity among patients with hip fractures when compared to those who are not vaccinated. Furthermore, it decreased the risk of acquiring COVID-19, and subsequent postoperative complications. Among those who are infected with COVID-19 despite vaccination, the mortality was significantly lesser.

## Introduction

Due to the growing population and a reversing age pyramid, the incidence of osteoporotic hip fracture is rising [1]. About 65000 people sustained hip fractures in the UK every year [2,3]. The management of hip fractures has been a major economic burden globally owing to frailty requiring longer hospital stays and rehabilitation [4].

The first case of coronavirus disease 2019 (COVID-19) infection caused by severe acute respiratory syndrome coronavirus 2 (SARS‑CoV‑2) was reported in December 2019 in Wuhan, Hubei Province of China. The virus subsequently spread throughout the world and was declared a public health emergency of international concern by the WHO on 30 January 2020 [5]. Countries around the world had planned lockdowns and social distancing to try and limit the spread of the virus. The UK restricted non-essential contact and travel on 16 March 2020. Epidemiological studies from that time showed, though there was a reduction in the overall trauma admissions, the rate of fragility fractures in the elderly remained constant. Studies have shown that contracting COVID infection during the peri-operative period increases mortality in hip fracture patients [6-8]. The 30-day post-operative mortality rate of hip fractures in the UK was about 6.5% in 2019 in the pre-COVID times. The 30-day mortality in hip fracture patients rose to about 8.3% at the end of 2020 [3]. However, multiple studies have indicated that mortality is much higher (30-40%) in those who have had hip fractures with concomitant COVID-19 infection [3,6-9].

In the early 2020s, countries around the world made a major commitment to developing a vaccine against SARS‑CoV‑2 resulting in public vaccination drives by December 2020 [10]. As part of the COVID-19 vaccination program, Pfizer-BioNTech's messenger RNA (mRNA) vaccine, BNT162b2, was approved for use in the UK in December 2020. Following that, OxfordAstraZeneca was also approved. Vaccination reduced the spread of the infection and prevented severe illness from the virus decreasing mortality in the elderly population [11,12]. Hip fractures commonly occur in elderly patients who tend to have multiple co-morbid conditions [13]. These are patients who are at extremely high risk of mortality during the COVID-19 pandemic, especially due to the disruption of the healthcare system. We don’t yet know if being vaccinated against COVID-19 by itself has any impact on post-operative mortality and morbidity in hip fracture patients.

The primary aim of our study was to evaluate the impact of COVID-19 vaccination on mortality in patients with hip fractures by comparing it to the control group without vaccination. Our secondary objective was to compare the impact on post-operative morbidity parameters like length of hospital stay and complications.

## Materials and methods

This is a retrospective case-control study involving 619 patients with hip fractures who underwent surgery in our tertiary care hospital between March 2020 and December 2021. Study participants included those defined as ‘hip fractures’ by the National Hip Fracture Database, which included intracapsular neck of femur, intertrochanteric, and subtrochanteric fractures. Those who had open fractures, isolated greater trochanter fractures, Shaft/distal femur fractures, pelvic fractures, or periprosthetic fractures were excluded. We excluded patients who had pathological fractures or polytrauma patients, which could by itself impact mortality/morbidity. Those patients who were managed with nonoperative measures and those unfit for surgery were also excluded. The study was registered in the audit and research department of the trust and ethical approval was taken.

The data were collected by accessing the health care information (HIS) system which stores the patient electronic medical records for the hospital. Data pertaining to the patient demographics (age, sex), type of fracture, the surgical procedure, Nottingham hip fracture score, ASA grade, type of anaesthesia used and abbreviated mental test score (AMTS) was obtained. Preoperative comorbidities were assessed by calculating Charlson Comorbidity Index. An RT-PCR test of respiratory tract samples that resulted in a positive result, clinical symptoms of COVID-19, or chest abnormalities on computed tomography (CT) that indicated COVID-19 were diagnostic criteria for COVID-19 infection.

All the patients were considered in the “Vaccinated group” if they had received two doses of the approved vaccine (Pfizer BioNTech or the Oxford-AstraZeneca) at least 14 days before getting admitted with a hip fracture and the remaining patients were considered in the ‘Control or Non-Vaccinated’ group. We compared mortality rates and morbidity parameters between the vaccinated group and the control group. Our primary outcome measures were 30- and 90-day mortality. Secondary outcome measures included post-operative complications like thromboembolic complications, cardiac, respiratory complications, etc. Mortality among the COVID-positive patients was also compared between the groups.

Statistical analysis

Data were entered in an Excel sheet (Microsoft Corporation, Redmond, WA) and analyzed using SPSS v22 software (IBM Corp., Armonk, NY). Data are represented in suitable tabular form. Continuous data are presented as mean and standard deviation. Categorical data are presented as percentages. The difference in means between the two groups was compared using the student t-test and the difference in proportion between the two groups was compared by the chi-square test. The Cox regression model was used for the comparison of survival among various groups. A p-value of <0.05 was considered significant.

## Results

We present the results of the first report on the effect of COVID-19 vaccination on post-operative mortality and morbidity in hip fracture patients. A total of 619 patients who had an operative intervention for hip fractures during the study period were included for final interpretation. Three hundred patients who had at least two doses of an approved vaccine were included in the vaccination group, whereas 319 patients who were not vaccinated were included in the control group. There were no significant differences in the baseline demographic parameters like age, sex, types of fractures, and type of surgery between the two groups. The mean Nottingham Hip Fracture Score and AMTS were also similar between the groups. However, the Charlson Comorbidity Index was higher in the vaccinated group with a significant number having had a previous myocardial infarction or congestive heart failure when compared to the control group. Despite having a higher baseline prevalence of cardiac comorbidities, the vaccinated group had fewer post-operative cardiac, thromboembolic, and neurological complications(p>0.05). Moreover, electrolyte imbalance and acute kidney injury (AKI) were also seen in fewer patients in the vaccinated group compared to the control group (p<0.05) (Table 1).

**Table 1:** Comparison of various parameters between the vaccinated and control groups * P-value <0.05 was considered statistically significant. ASA: American Society of Anesthesiologists; AMTS: abbreviated mental test score; CKD: chronic kidney disease; DVT: deep vein thrombosis; AKI: acute kidney injury; CHF: congestive heart failure; PVD: peripheral vascular disease; CVA: cerebrovascular accident; COPD: chronic obstructive pulmonary disease

Parameters	Number of cases	Not vaccinated (n-319)			Vaccinated (n-300)			P-value
		n	%		n		%	
Total no. of patients	619	319			300			0.92
Age								
(Mean + SD) yrs	619	79.31 + 11.24			80.59 + 9.40			0.124
Sex								
Female	412	214	67.1%		198		66.0%	0.775
Male	207	105	32.9%		102		34.0%	
ASA								
1	8	5	1.5%		3		1%	0.342
2	80	38	11.9%		42		14%	
3	451	230	72.1%		221		73.6%	
4	82	46	14.6%		35		11.6%	
Type of fracture								
Intracapsular fracture	353	182	56.2%		171		58.0%	0.546
Extracapsular fracture	266	137	43.8%		129		42.0%	
Nottingham Hip Fracture Score								
Mean + SD	619	4.77 + 1.68			4.81 + 1.60			0.913
AMTS								
Mean + SD	619	7.02 + 3.61			6.93 + 3.69			0.770
Length of stay								
(Mean + SD) days	619	12.87 + 9.47			13.32 + 8.89			0.544
COVID status								
Negative	555	271		85.0%	280	93.3%		<0.001*
Positve	68	48		15.0%	20	6.7%		
Mortality rates								
30 days mortality	59	42		13.2%	17	5.7%		<0.001*
90 days mortality	123	79		24.8%	44	14.7%		0.002
Complications								
DVT/embolism	17	13		4.1%	4	1.3%		0.137
Respiratory complication	128	70		21.9%	58	19.3%		0.423
Cardiac complication	58	33		10.3%	25	8.3%		0.216
Neurologic complication	18	11		3.4%	7	2.3%		0.812
Electrolyte imbalance	142	88		27.6%	54	18.0%		<0.001
AKI	84	56		17.5%	28	9.3%		<0.001
Other	52	20		6.1%	32	10.6%		0.443
Charlson Comorbidity Index								
(Mean + SD)	619	4.83 + 2.21			5.57 + 2.08			<0.001
Myocardial infarction	139	49		15.4%	90	30.0%		<0.001
CHF	127	53		16.6%	74	24.7%		0.013
PVD	9	7		2.2%	2	0.7%		0.113
CVA	90	45		14.1%	45	15.0%		0.753
Dementia	197	100		31.3%	97	32.3%		0.793
COPD	106	55		17.2%	51	17.0%		0.937
Connective tissue disease	23	3		0.9%	20	6.7%		<0.001
Peptic ulcer	11	8		2.5%	3	1.0%		0.156
Liver disease	8	6		1.9%	2	0.7%		0.181
Diabetes	110	55		17.2%	55	18.3%		0.722
Hemiplegia	21	8		2.5%	13	4.3%		0.210
CKD	48	17		5.3%	31	10.3%		0.020
Tumour	93	42		13.2%	51	17.0%		0.182
AIDS	24	0		0.0%	24	8.0%		<0.001

The 30-day post-operative mortality score was significantly higher at 13.2% in the unvaccinated group when compared to 5.3% in the vaccinated group (p <0.05). Similarly, the 90-day mortality was also higher in the unvaccinated group at 24.8% when compared to 14.7% in the unvaccinated group (p <0.05). The incidence of COVID-19 infection during hospital admission in the perioperative period was 6.6%. This was significantly higher in the unvaccinated group, with 15% of the patients getting the COVID-19 infection during their perioperative period (p<0.001). Furthermore, it was found that among the vaccinated patients who did, unfortunately, have perioperative COVID-19 infection, the 30-day mortality was significantly lower (10%) compared to the control group (31.2%) (p 0.001). Similarly, the 90- day mortality was significantly lower (25%) compared to the control group (43.75%) (p<0.001) (Table 2).

**Table 2:** Comparison of 30-day and 90-day mortality among COVID-positive between the vaccinated and non-vaccinated

Parameters	Total no. of cases	COVID-positive among the not vaccinated (n=48)		COVID-positive among the vaccinated (n=20)		P-value
		n	%	n	%	
30-day mortality among COVID positive	17	15	31.2%	2	10.0%	<0.001
90-day mortality among COVID positive	26	21	43.75%	5	25.0%	<0.001

Cox regression analysis of 30-day survival adjusted with age, sex, NHFS, COVID status and ASA depicted that vaccination independently decreased mortality among patients with hip fractures. Similar results were also seen for 90-day mortality rates (Tables 3, 4)

**Table 3:** Cox regression model for 30 days survival according to vaccination status ASA: American Society of Anesthesiologists; AMTS: abbreviated mental test score; NHF: National Hip Fracture

Predictors in model	Hazard ratio	95% CI		P-value
		Lower	Upper	
Age, sex and vaccination status				
No	Reference	-	-	-
Yes	0.397	0.226	0.698	0.001
NHF score, ASA grade and vaccination status				
No	Reference	-	-	-
Yes	0.504	0.279	0.911	0.023
COVID-19 status, AMTS, Charlson Comorbidity Index and vaccination status				
No	Reference	-	-	-
Yes	0.369	0.208	0.654	0.001

**Table 4:** Cox regression model for 90 days survival according to vaccination status ASA: American Society of Anesthesiologists; AMTS: abbreviated mental test score; NHF: National Hip Fracture

Predictors in model	Hazard ratio	95% CI		P-value
		Lower	Upper	
Age, sex and vaccination status				
No	Reference	-	-	-
Yes	0.523	0.361	0.758	0.001
NHF score, ASA grade and vaccination status				
No	Reference	-	-	-
Yes	0.608	0.414	0.894	0.011
COVID-19 status, AMTS, Charlson Comorbidity Index and vaccination status				
No	Reference	-	-	-
Yes	0.511	0.351	0.745	<0.001

## Discussion

A majority of the UK population continues to be affected by COVID-19, and evidence of vaccine effectiveness is essential for policy decisions regarding the continued delivery of the program and other non-drug interventions [14]. According to preliminary results from phase III clinical trials, BNT162b2 and ChAdOx1-S vaccines are highly effective when administered in two doses at three-week and four-week intervals, respectively [15,16]. However, some studies have suggested that the vaccine is less effective in elderly frail patients [17]. Hip fractures are commonly seen in elderly frail patients and are associated with high mortality when infected with concomitant COVID-19 [18-20]. The effectiveness of the vaccine in hip fractures may be crucial in reducing the mortality among these patients but there is no evidence depicting the same at present. We aimed to evaluate the effectiveness of COVID-19 vaccination among patients with hip fractures in reducing mortality and morbidity when compared to the control group who were not vaccinated.

While the groups were not matched, they shared similar baseline characteristics such as age, sex, NHFS, ASA, fracture type and surgery. However, the Charlson Comorbidity Index was high in the vaccinated group compared to the control group (p<0.001). As part of the first phase of the vaccination program, older residents of care homes and their carers, people aged 80 years and older and frontline healthcare workers were prioritized for vaccination. A vaccine program was later expanded to reach those over 70 years of age and clinically extremely vulnerable individuals [14]. This explains the high CCI among the vaccinated, as these are the ones who were prioritized in the initial phases of the vaccination program.

A prospective study done by Anwar et al. with cancer patients scheduled for elective surgeries showed that the mortality and morbidity were significantly lower in the vaccinated group when compared to the unvaccinated group [21]. A multicentric, collaborative study involving 1128 patients looking at mortality and pulmonary complications, also collaborative, demonstrated that pre-operative SARS-CoV-2 vaccination led to reduced risk of both postoperative mortality and pulmonary complications in SARS-CoV-2-positive surgical patients [22]. The 30-day and 90-day mortality among the vaccinated groups was significantly lower when compared to the control group. Moreover, the number of people getting infected with COVID-19 was also less in vaccinated individuals. The results are significant as they demonstrate that vaccination not only reduced the risk of postoperative mortality in hip fracture patients but also reduced the perioperative risk of contracting the COVID-19 infection. This was despite the fact that the vaccinated group had higher CCI and was frailer. Multivariate regression analysis adjusting for various demographic variables and comorbidities also demonstrated that vaccination independently improved survival among hip fractures.

According to Moghadas et al., if a vaccinated patient contracts the infection, the clinical severity is not very bad due to circulating antibodies. They demonstrated that vaccination significantly reduces complications requiring lesser intensive care admissions [23]. Several studies during COVID-19 times have shown that pulmonary complications are significantly high in the post-operative period [22,24]. In our study, the complications, especially AKI and electrolyte balance, were significantly lesser in the vaccination group. Though the pulmonary complications were less the results were not statistically significant. However, there was no difference in the length of hospital stay.

In the systematic review to determine the mortality of concomitant infection of COVID-19 with hip fractures, Mastan et al. showed the 30-day mortality was 32.5% in COVID-positive patients compared to 8.3% in COVID-negative patients [20]. But the mortality rates among vaccinated patients are not available in the literature. In our study, the 30-day mortality among vaccinated COVID-positive patients was significantly lower at 10% when compared to 31.2% in the control group. The improved mortality rate in the vaccination group with concomitant COVID-19 infection is due to lesser complications and better utilization of hospital resources in them.

Our study has some limitations. The effect of multiple variants of coronavirus like the Delta variant, Omicron, etc. has not been considered. Furthermore, the effect of innate immunity acquired by patients who are already infected with COVID-19 on vaccination is also not considered. Also, it may be recommended to investigate the comparison of the impact of a single dose, double dose, or booster doses of COVID-19 vaccination, which was beyond the scope of this study. A better understanding of the COVID-19 disease and better utilization of hospital resources, social distancing, etc. also contributed to improved mortality and morbidity in the post-vaccination times but the impact of these is difficult to quantify. Nevertheless, this is the first study that investigates the impact of vaccination on patients with hip fractures and clearly demonstrates the improved mortality and morbidity in them.

## Conclusions

Vaccination against COVID-19 independently reduced mortality and morbidity among patients with hip fractures when compared to those who are not vaccinated. Furthermore, it decreased the risk of acquiring COVID-19 and subsequent postoperative complications. Among those who are infected with COVID-19 despite vaccination, mortality was significantly lesser.
